# Demographic and Treatment Analysis of Periosteal Osteosarcoma

**DOI:** 10.1002/cnr2.70086

**Published:** 2024-12-15

**Authors:** Michael C. Larkins

**Affiliations:** ^1^ Brody School of Medicine (BSOM) East Carolina University (ECU) Greenville North Carolina USA; ^2^ Department of Emergency Medicine Boonshoft School of Medicine at Wright State University Fairborn Ohio USA

**Keywords:** cancer epidemiology, chemotherapy, periosteal osteosarcoma, surgical oncology

## Abstract

**Background and Aims:**

Periosteal osteosarcoma (PO) is a rare bone cancer that makes up between 1% and 6% of osteosarcomas. No epidemiological survey of the United States has been conducted to study this disease, and most of the literature is limited to single‐center analyses and case reports. We seek to perform the first such assessment.

**Methods:**

The Surveillance, Epidemiology, and End Results (SEER) Program was queried for patients with primary PO (ICD‐O‐3 code 9193/3). Analysis of demographic, disease, and treatment variables was conducted via Fisher's exact test and 20‐year cause‐specific survival (20y CSS) was assessed via logrank analysis.

**Results:**

Fifty‐four patients with PO were identified; median age was 20–24 years at diagnosis. Multivariate analysis demonstrated surgery provided 20y CSS benefit (hazard ratio [HR] = 0.08, *p* = 0.040) while chemotherapy (CTX) did not (*p* = 0.29); however, given the limited number of events (*n* = 11), recalculation of Cox regression for each variable demonstrated significance only with race (*p* = 0.026). Younger patients were more likely to be diagnosed with PO of the appendicular skeleton compared to the axial skeleton (*p* = 0.038). Mean survival time was greater among patients diagnosed with appendicular PO (16.0 years [14.2, 17.9]) compared to axial PO (10.9 years [9.5, 12.4]). Stage‐stratified survival analysis demonstrated surgery alone was non‐inferior to surgery with the addition of CTX (local disease: *p* = 0.37; regional disease: *p* = 0.85).

**Conclusion:**

Axial PO is associated with decreased mean 20y CSS compared to appendicular PO, though in general, appendicular PO is more common than axial PO. In keeping with current literature, treatment of PO with CTX in addition to surgery should be reserved for high‐risk patients, though its use in the treatment of PO is questionable.

## 1 Introduction

Periosteal osteosarcoma (PO) is a rare sarcoma that makes up 1%–6% of all osteosarcomas [1]. The overall incidence of osteosarcoma in the United States has been reported as 0.2 to three per 100 000 malignant bone tumors [2]. Comparatively, osteosarcomas in general are the most common primary osseous sarcoma, with an incidence of 5 per 1 million patients among patients less than 19 and 3.5 per 1 million for patients older than 60 years [3]. PO has been reported as occurring most frequently in the second and third decades of life, and most frequently affecting the tibia, femur, ulna, and humerus [4]. Among patients with osteosarcoma (not specifically PO), 1‐, 5‐, and 10‐year survival has been reported as higher among those diagnosed with appendicular as opposed to axial skeletal cancer [3], though a similar analysis has yet to be conducted regarding PO.

While no guidelines regarding the treatment and management of PO exist, the general consensus derived from scant literature suggests surgery is the most effective management strategy for most patients, with chemotherapy (CTX) being reserved for high‐risk patients [5]. Comparatively, osteosarcomas, in general, are managed with surgical resection, reconstruction, and both neoadjuvant and adjuvant CTX [3]. Ten‐year overall survival rates of PO have been reported at 84% [6], while comparatively, osteosarcomas, in general, have been reported to have 5‐year survival rates of 70% for those with local disease at diagnosis and 20% for those with metastatic or recurrent disease [3]. Despite the high survival rates reported among those with PO, no longitudinal study of survival (e.g., greater than 10 years) has been conducted at the time of writing. To date, analysis of PO in the literature has been limited to single‐center analyses [1, 7, 8, 9] and case reports [10, 11], with one multicenter study having been conducted in Europe in 2005 [12]; no epidemiological survey of the United States or SEER has been conducted over the past 20 years. This study seeks to fill this gap with longitudinal, cause‐specific survival data and provide an analysis of demographic and treatment factors that may improve the already high reported survival rate of this disease.

## 2 Methods

### 2.1 Patient Selection

The National Cancer Institute's Surveillance, Epidemiology, and End Results (SEER) Program was queried to identify patients with a diagnosis of PO. The 17‐registry Incidence data set with cases diagnosed between 2000 and 2020 was selected [13]. Inclusion criteria were International Classification of Diseases (ICD)‐O‐3 histology/behavior code 9193/2 corresponding to PO and first malignant primary indicator = “Yes.” Surgical codes were determined based on the 2021 SEER Staging Manual [14].

### 2.2 Data Analysis

Data was analyzed in SPSS (Version 29.0; Armonk, NY: IBM Corp.), with a *p* < 0.05 as the cutoff for statistical significance and 95% confidence intervals reported in brackets [CI]. Categorical variables were compared via a two‐sided Fisher's exact test. Kaplan–Meier (KM) survival curves were generated and compared using logrank analysis based on 20‐year cause‐specific survival (20y CSS); multivariate analysis was performed using Cox regression.

## 3 Results

### 3.1 Cohort Characteristics

**FIGURE 1:**
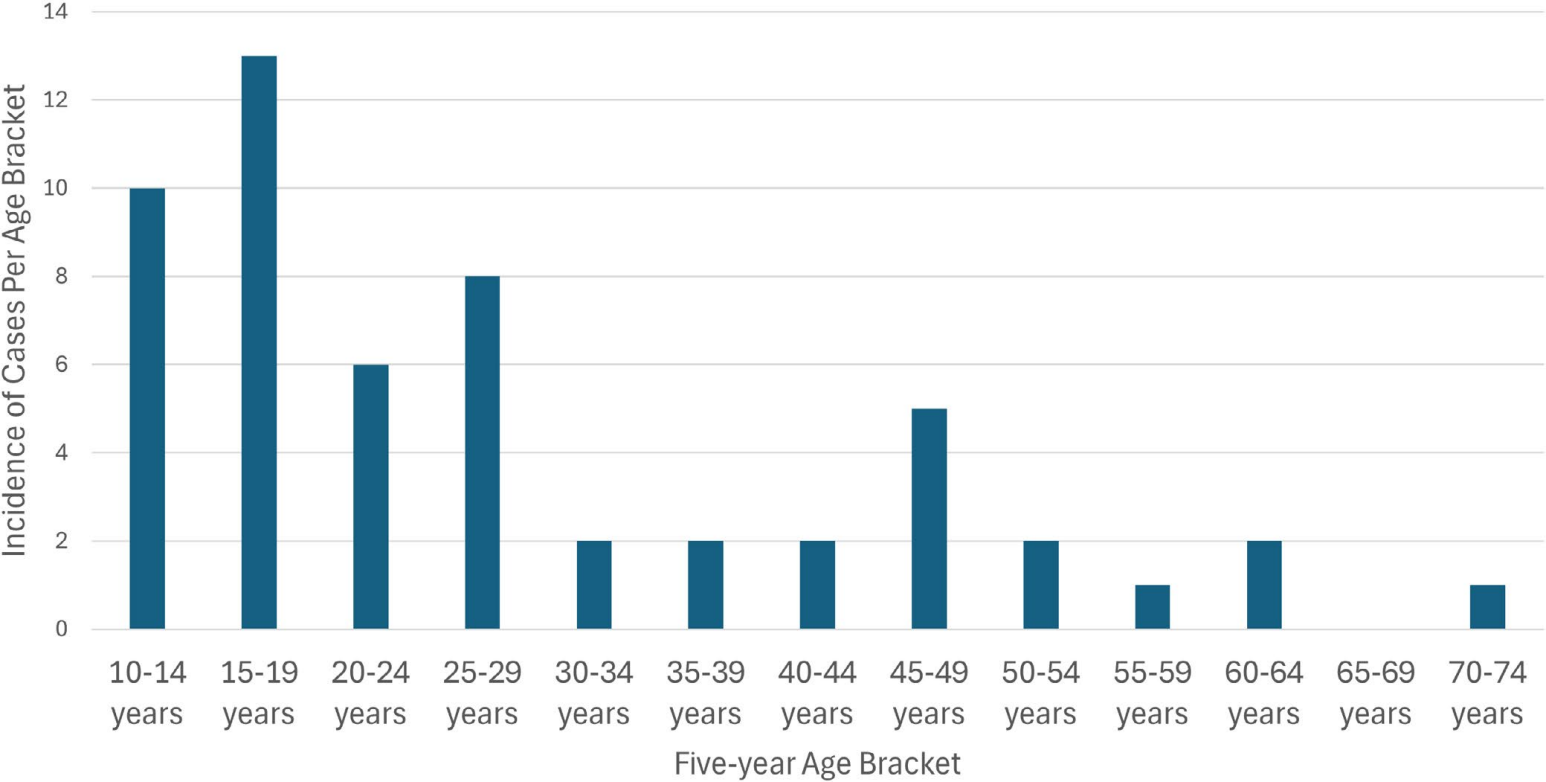
Age at diagnosis of periosteal osteosarcoma. Histogram depicting the age at diagnosis of periosteal osteosarcoma for patients identified between 2000 and 2020.

Fifty‐four patients with primary PO were identified. All patients had complete survival information, all cases had disease classified as malignant, and all patients were treated within 3 months of diagnosis. Median age bracket was 20–24 years of age at diagnosis. Age was split into bivariate groups to allow for strong statistical comparison, though a histogram displaying the age distribution of diagnoses can be found in Figure 1. One patient had a disease diagnosed radiographically without microscopic confirmation and one other patient had a disease diagnosed via exfoliative cytology; all others (*n* = 52) had histologically confirmed disease. Demographic and disease characteristics can be found in Table 1.

**TABLE 1:** Demographic and disease characteristics of patients diagnosed with periosteal osteosarcoma in the United States between 2000 and 2020.

Variable	Number (% of cohort; *n* = 54)
Age at diagnosis	
10–24 years	29 (53.7%)
25–74 years	24 (46.3%)
Sex	
Male	35 (64.8%)
Female	19 (35.2%)
Race	
American Indian	1 (1.9%)
Asian/Pacific Islander	6 (11.1%)
Black	10 (18.5%)
White	37 (68.5%)
Summary stage	
Local	27 (50.0%)
Regional	15 (27.8%)
Distant	3 (5.6%)
Unstaged/incomplete staging	9 (16.7%)
Grade	
I (Well‐differentiated)	3 (5.6%)
II (Moderately differentiated)	12 (22.2%)
III (Poorly differentiated)	10 (18.5%)
IV (Undifferentiated)	13 (24.1%)
Unknown/incomplete grading	16 (29.6%)
Axial vs. appendicular skeleton	
Axial	9 (16.7%)
Appendicular	45 (83.3%)
Laterality	
Left	23 (42.6%)
Right	28 (51.9%)
N/A	3 (5.6%)

*Note:* Demographic and disease characteristics for patients diagnosed with periosteal osteosarcoma identified via the SEER Program. The appendicular skeleton was considered primary site codes C40.0 (long bones of the upper limb) and C40.2–C40.3 (long and short bones of the lower limb), and the axial skeleton was considered as primary site codes C41.1 (mandible), C41.3 (rib, sternum, clavicle), and C41.4 (pelvic bones, sacrum, coccyx).

Treatment information regarding the identified group of patients can be found in Table 2. Three patients were listed as having one to three lymph nodes removed in addition to undergoing surgical treatment. Patients were also stratified by skeletal location of PO for further analysis. This can be found in Table 3. In general, it can be seen that appendicular PO occurs somewhat more commonly among younger patients (10–54 years of age at diagnosis).

**TABLE 2:** Treatment characteristics of patients diagnosed with periosteal osteosarcoma in the United States between 2000 and 2020.

Variable	Number (% of Cohort; *n* = 54)
Treatment	
No treatment	3 (5.6%)
Surgical treatment only	28 (51.9%)
Surgery with adjuvant radiotherapy (RT)	1 (1.9%)
Chemotherapy (CTX) only	2 (3.7%)
Surgery + neoadjuvant CTX	4 (7.4%)
Surgery + adjuvant CTX	8 (14.8%)
Surgery + neo‐ and adjuvant CTX	7 (13.0%)
Surgery, CTX, RT	1 (1.9%)
Surgical procedure	
None	5 (9.3%)
Local tumor destruction/partial resection	9 (16.7%)
Radical surgery with limb salvage	35 (64.8%)
Major amputation	4 (7.4%)
Surgery not otherwise specified	1 (1.9%)
CTX given	
Yes	33 (61.1%)
No	21 (38.9%)

*Note:* Treatment characteristics for patients diagnosed with periosteal osteosarcoma identified via the SEER Program. Surgical codes were determined based on the SEER 2021 Staging Manual Appendix C: Surgery Codes [14]. Local surgery and partial resection procedures were based on codes 15–26, radical surgery with limb salvage corresponded to code 30, and codes 51–54 were classified as major amputation. Information on the specific chemotherapeutic regimen administered was not available.

**TABLE 3:** Clinicopathologic and treatment characteristics for patients diagnosed with periosteal osteosarcoma stratified by skeletal location.

Variable	Axial	Appendicular
	Number (% of axial cases)	Number (% of appendicular cases)
Age		
10–24 years	2 (22.2%)	27 (60.0%)
25–74 years	7 (77.8%)	18 (40.0%)
Sex		
Male	4 (44.4%)	15 (33.3%)
Female	5 (55.6%)	30 (66.7%)
Race		
American Indian	0 (0.0%)	1 (2.2%)
Asian/Pacific Islander	1 (11.1%)	5 (11.1%)
Black	1 (11.1%)	9 (20.0%)
White	7 (77.8%)	30 (66.7%)
Grade		
I	2 (22.2%)	1 (2.2%)
II	2 (22.2%)	10 (22.2%)
III	2 (22.2%)	8 (17.8%)
IV	3 (33.3%)	10 (22.2%)
Unknown	0 (0.0%)	16 (35.6%)
Laterality		
Left	1 (11.1%)	22 (48.9%)
Right	5 (55.6%)	23 (51.1%)
N/A	3 (33.3%)	0 (0.0%)
Treatment		
No treatment	0 (0.0%)	3 (6.7%)
Surgical treatment only	3 (33.3%)	23 (51.1%)
Surgery with adjuvant radiotherapy (RT)	0 (0.0%)	1 (2.2%)
Chemotherapy (CTX) only	1 (11.1%)	1 (2.2%)
Surgery + neoadjuvant CTX	0 (0.0%)	5 (11.1%)
Surgery + adjuvant CTX	3 (33.3%)	5 (11.1%)
Surgery + neo‐ and adjuvant CTX	2 (22.2%)	6 (13.3%)
Surgery, RT, CTX	0 (0.0%)	1 (2.2%)
Surgical procedure		
None	1 (11.1%)	4 (8.9%)
Local tumor destruction/partial resection	2 (22.2%)	7 (15.5%)
Radical surgery with limb salvage	4 (44.4%)	31 (68.9%)
Major amputation	1 (11.1%)	3 (6.7%)
Surgery not otherwise specified	1 (11.1%)	0 (0.0%)
CTX given		
Yes	6 (66.7%)	27 (60.0%)
No	3 (33.3%)	18 (40.0%)

*Note:* Relevant demographic, disease, and treatment characteristics of patients diagnosed with periosteal osteosarcoma stratified by skeletal location (axial vs. appendicular skeleton). Axial cases (*n* = 9) made up 16.7% of the cohort, appendicular cases (*n* = 45) made up 83.3% of the cohort.

### 3.2 Multivariate Analysis

Cox regression analysis demonstrated increased 20y CSS among patients treated surgically compared to those who did not (hazard ratio [HR] = 0.08, *p* = 0.040); all other variables did not demonstrate an impact on survival. It should be noted that only 11 events were observed over the analysis period among the 54 patients identified. Given this, Cox regression analysis was performed with each individual variable a second time. Results can be found in Table 4.

**TABLE 4:** Individual Cox regression analysis of 20‐year cause‐specific survival among patients with periosteal osteosarcoma (PO).

Variable	Hazard ratio	*p*
10–24 years/25–74 years^a^	1.91	0.33
Male/female	0.54	0.42
Race (American Indian vs. Asian vs. Black vs. White)	N/A	0.026
Axial/appendicular skeletal location	33.27	0.35
Laterality (left vs. right vs. N/A)	N/A	0.94
Grade (I vs. II vs. III vs. IV vs. unknown)	N/A	0.53
Surgical treatment Y/N	1.07	0.95
Chemotherapy Y/N	0.45	0.33

*Note:* Results from Cox regression analysis performed for each variable identified individually. Overall *p* value is listed for categorical variables with more than two categories.

^a^ Age at diagnosis with PO.

### 3.3 Prognostic/Disease Trends Based on Age

**FIGURE 2:**
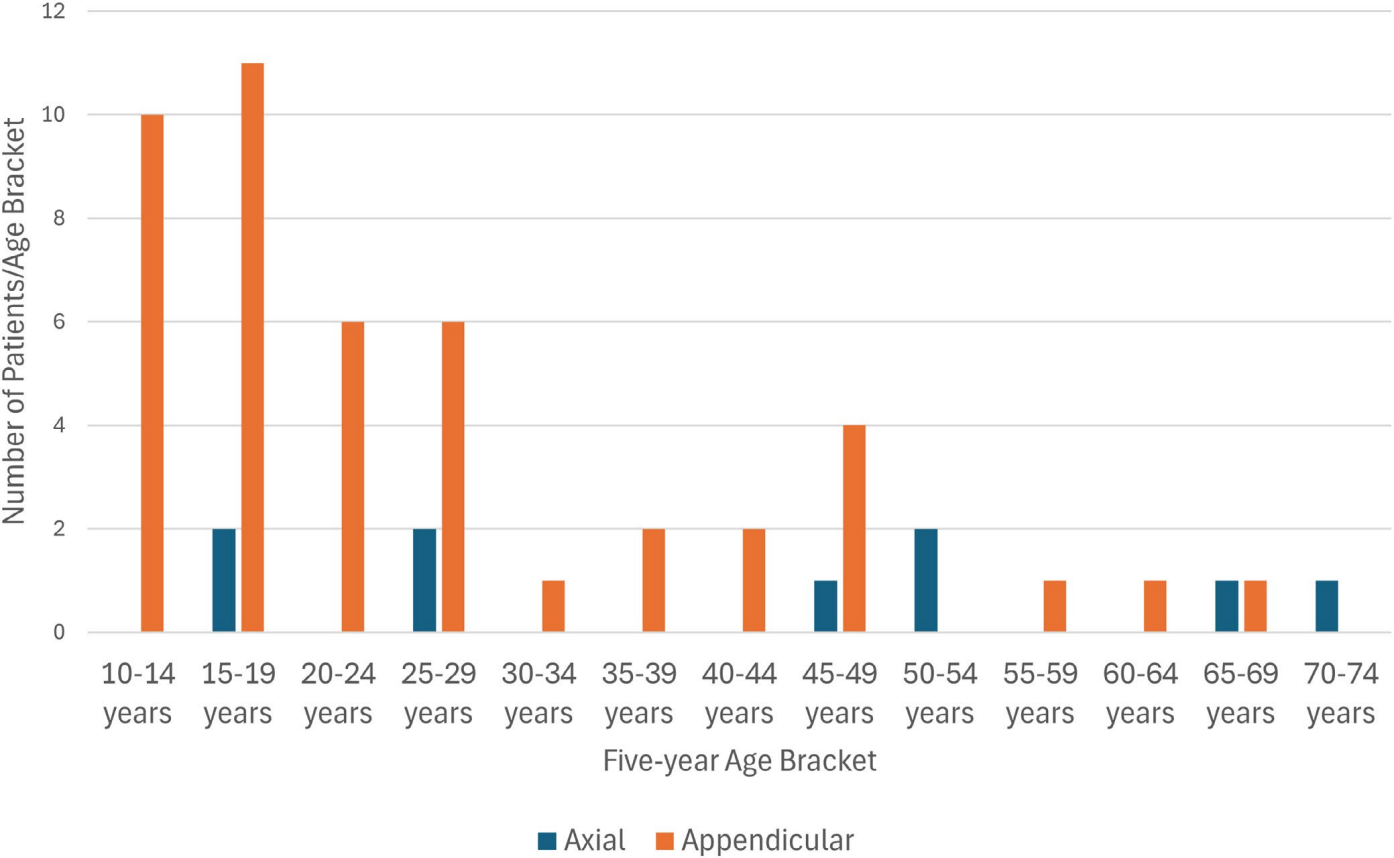
Frequency of periosteal osteosarcoma (PO) diagnosis based on patient age. The frequency of PO is stratified by skeletal location (appendicular vs. axial) and patient age (as reported in 5‐year increments from the SEER program). A total of 73% of patients diagnosed with appendicular PO were < 30 years at diagnosis compared to 44% of the patients with axial PO diagnosed at < 30 years of age.

Based on the histogram in Figure 1, it can be seen that cases of PO are more frequently diagnosed among patients < 30 years of age. Younger patients diagnosed with PO are more likely to be diagnosed with disease in the appendicular skeleton (*p* = 0.038; see Figure 2). No patients were diagnosed with PO of the axial skeleton younger than 15 years, while the oldest patient in this entire cohort had PO of the axial skeleton (specifically of the ribs or sternum; the patient age bracket was 70–74 years). No difference in the relative proportion of patients diagnosed with PO with respect to age and sex (*p* = 0.45), age and race (*p* = 0.39), age and disease stage (*p* = 0.87), or age and grade (*p* = 0.32) was observed. No difference in the frequency of CTX among patients with low‐grade (Grade I or II; 15 patients) PO was seen compared to high‐grade (Grade III or IV; 23 patients) were appreciated (*p* = 0.17).

**FIGURE 3:**
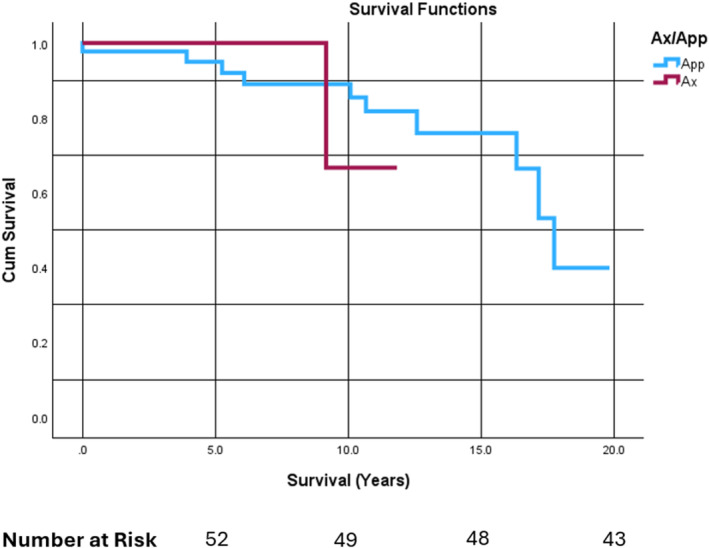
Cause‐specific survival (CSS) of patients with periosteal osteosarcoma (PO) stratified by skeletal location. Twenty‐year CSS of patients with PO stratified by skeletal location, with “App” referring to PO of the appendicular skeleton and “Ax” referring to PO of the axial skeleton. Mean survival time among those with axial PO (10.9 years [9.5, 12.4]) was lower than among those with appendicular PO (16.0 years [14.2, 17.9]). Appendicular PO 20y CSS was 39.9% [7.7%, 72.1%], while axial PO survival was 66.7% [12.3%, 100.0%]. Logrank comparison was nonsignificant (*p* = 0.78). The number at risk over time is listed below the primary *x*‐axis.

No 20y CSS difference was appreciated between patients diagnosed with PO of the axial skeleton (66.7% [12.3%, 100.0%]) versus the appendicular skeleton (39.9% [7.7%, 72.1%]; *p* = 0.78), though mean survival time among those with axial PO (10.9 years [9.5, 12.4]) was lower than among with appendicular PO (16.0 years [14.2, 17.9]). A KM comparison between skeletal locations of PO can be seen in Figure 3.

### 3.4 Stage‐Stratified Treatment Subanalysis

Analysis of treatment outcomes was conducted with respect to disease stage; patients with incomplete staging information were excluded (*n* = 9). No difference was seen among patients with local PO with respect to surgical treatment (Y/N; *p* = 0.63); one patient with local disease did not undergo surgical management and thus mean survival time could not be calculated. Those with regional disease that underwent surgery (57.1% [17.3%, 96.9%]) had decreased 20y CSS compared to those that did not (66.7% [12.3%, 100.0%]; *p* = 0.046). Mean survival time among those that underwent surgery with regional disease was 14.4 years [12.2, 16.6] compared to 1.0 years for those that did not [0.2, 1.8]. All patients with local disease who did not undergo surgical treatment died within 12 years of diagnosis; this number dropped to 2 years among those with regional disease. Analysis was limited among those with distant disease, as all three of these patients underwent surgical treatment and no comparison among those that did not receive surgical treatment was possible.

## 4 Discussion

In this analysis of PO among patients in the United States diagnosed between 2000 and 2020, 54 patients with primary PO were identified. Among these patients, 96% had histologically confirmed disease, with the remainder having been diagnosed radiographically or with exfoliative cytology. Only surgical treatment was associated with increased 20y CSS during Cox regression analysis (*p* = 0.040), though adjusting for the limited number of events seen during the 20‐year survival period, only patient race was seen to be significant (*p* = 0.026). However, a subanalysis of patients based on age found that younger patients were more likely to be diagnosed with PO, particularly if they were under the age of 30 years. Furthermore, younger patients were more likely to be diagnosed with PO of the appendicular skeleton (*p* = 0.038) compared to PO of the axial skeleton. Axial PO was associated with decreased mean survival time, though 20y CSS did not demonstrate significance (*p* = 0.78).

To date, the largest study of PO the author of this article found was a 120‐patient study from the Rizzoli Institute in 2005 by Bertoni et al. [9]. This study was focused mainly on dedifferentiated PO, a variant of osteosarcoma thought to coexist with conventional PO (the type studied in this article); thus, only 29 of the 120 patients were analyzed and only one of this cohort of 29 with dedifferentiated PO had axial PO (PO of the pelvis). Dedifferentiated PO was considered more malignant than conventional PO, and thus, wide local excision with/without CTX was recommended for treatment in general. The second largest study (and largest in terms of raw cases of PO) was Grimer et al., a European analysis of the European Musculo Skeletal Oncology Society (EMSOS) database [12]. While two‐thirds of the patients in this analysis received CTX, no difference in survival was seen with treatment with CTX.

The most recent article focused on PO outcomes, Alpan et al., was a single‐center study composed of 27 patients, all of whom had PO of the appendicular skeleton [1]. This study focused on prognostic factors important for survival and was mainly an exploration of surgical factors. Disease characteristics such as medullary involvement and nondelayed diagnosis with needle biopsy at surgical resection were recommended. CTX was reserved for high‐grade or misdiagnosed patients. Six patients were identified in this study as having a delay in treatment greater than 6 months; comparatively, all patients in this analysis were treated within 3 months of diagnosis, potentially improving outcomes and reducing the frequency of CTX used in conjunction with surgery.

In comparison, intramedullary osteosarcoma is the most common osteosarcoma and the most common primary malignant bone tumor [15]. The majority of intramedullary osteosarcomas are high‐grade, with the current established treatment being surgery and systemic CTX. Neoadjuvant CTX is considered the most recently recommended treatment modality, though adjuvant CTX, when utilized, is employed with the goal of destroying remaining cancer cells after surgical resection. Comparatively, the 54 cases of PO in this analysis demonstrate an underutilization of CTX, with 33% of axial cases and 51% of appendicular cases being treated with surgery alone.

Analysis of patients receiving CTX only for treatment of PO was limited in this study (only two patients, 3.7%, underwent this treatment regimen). Furthermore, no benefit was seen based on the results of multivariate analysis. While few patients were treated with CTX alone, 61.1% of the entire cohort received CTX in combination with other therapies. Insufficient evidence exists both in the literature and in this analysis to support or disfavor the continued use of CTX, and continued incorporation of CTX, especially among patients with high‐grade disease or not amenable to surgical management, likely should still be considered.

The decreased survival associated with axial PO could be twofold: with axial PO occurring in patients across all ages, older patients with axial PO may have lower survival by virtue of their decreased resilience to the burden of treatment. Second, axial PO is likely more difficult to treat and may be more likely to spread to vital organs, given the close proximity. Wang et al. report a case of mandibular PO in a 39‐year‐old male, which ultimately recurred locally, requiring additional treatment [10]. Older or more frail patients may very well have not tolerated the initial surgery this patient underwent, let alone the revision. Do Le Hoang et al. report a case of clavicular PO treated with surgery and neoadjuvant CTX with a reportedly good outcome [11].

Key limitations of this study are its retrospective nature and small sample size. The rarity of PO compared to other osteosarcomas makes the study of epidemiological trends and the creation of guidelines difficult. As this analysis only identified 54 cases, stratification between various factors was challenging and, in some cases, not possible with sufficient statistical rigor. Furthermore, the SEER database is limited in the available information, particularly regarding the specifics of disease treatment and disease metastasis. More details regarding the chemotherapeutic regimens and the presence of medullary disease involvement were not available. In the same vein, detailed analysis and comparison between treatment modalities alone and in combination was limited given the limited granularity and epidemiologic nature of the SEER database [16, 17]. The database represents some 35% of the US population, and the literature has commented on cases of adjuvant CTX that are underreported in general.

Axial PO is more common among patients > 30 years old and is associated with decreased mean 20y CSS compared to appendicular PO, though in general, appendicular PO is more common than axial PO. In keeping with current literature, treatment of PO with CTX in addition to surgery should be reserved for high‐risk patients, though its utility in the treatment of PO remains unproven. Further analysis among a larger cohort and regarding the specifics of treatment is warranted. Specifically, further study of the utility of CTX could provide benefit to patients with PO and similar cancers.

## Author Contributions

**Michael C. Larkins:** conceptualization, investigation, writing – original draft, methodology, validation, writing – review and editing, formal analysis, project administration.

## Ethics Statement

All personal identifying information for patients was anonymized by Surveillance, Epidemiology, and End Results, so ethical approval and informed consent were waived.

## Consent

Consent for publication was waived due to approved use of publicly accessible database and retrospective nature of the study. This article does not contain any studies with human participants or animals performed by any of the authors.

## Conflicts of Interest

The author declares no conflicts of interest.

## Data Availability

The data and materials that supported the findings of this study are available from the corresponding author upon reasonable request. Original data are available at Surveillance Epidemiology and End Results (SEER) database (https://seer.cancer.gov/data/).
